# The Effects of Socioeconomic Vulnerability, Psychosocial Services, and Social Service Spending on Family Reunification: A Multilevel Longitudinal Analysis

**DOI:** 10.3390/ijerph14091040

**Published:** 2017-09-09

**Authors:** Tonino Esposito, Ashleigh Delaye, Martin Chabot, Nico Trocmé, David Rothwell, Sonia Hélie, Marie-Joelle Robichaud

**Affiliations:** 1 School of Social Work, University of Montreal, 3150, Jean-Brillant, Montreal, QC H3C 3J7, Canada; Sonia.helie@cjm-iu.qc.ca (S.H.); Marie-joelle.robichaud@umontreal.ca (M.-J.R.); 2 Centre for Research on Children and Families, McGill University, 3506, University Street, Montreal, QC H3A 2A7, Canada; Ashleigh.delaye@mail.mcgill.ca (A.D.); Martin.chabot@mcgill.ca (M.C.); Nico.trocme@mcgill.ca (N.T.); 3 School of Social and Behavioral Health Science, Oregon State University, 462, Waldo Hall, Corvallis, OR 97331, USA; david.rothwell@oregonstate.edu

**Keywords:** family reunification, socioeconomic vulnerability, jurisdictional effects, longitudinal, multilevel

## Abstract

Socio-environmental factors such as poverty, psychosocial services, and social services spending all could influence the challenges faced by vulnerable families. This paper examines the extent to which socioeconomic vulnerability, psychosocial service consultations, and preventative social services spending impacts the reunification for children placed in out-of-home care. This study uses a multilevel longitudinal research design that draws data from three sources: (1) longitudinal administrative data from Quebec’s child protection agencies; (2) 2006 and 2011 Canadian Census data; and, (3) intra-province health and social services data. The final data set included all children (*N* = 39,882) placed in out-of-home care for the first time between 1 April 2002 and 31 March 2013, and followed from their initial out-of-home placement. Multilevel hazard results indicate that socioeconomic vulnerability, controlling for psychosocial services and social services spending, contributes to the decreased likelihood of reunification. Specifically, socioeconomic vulnerability, psychosocial services, and social services spending account for 24.0% of the variation in jurisdictional reunification for younger children less than 5 years of age, 12.5% for children age 5 to 11 years and 21.4% for older children age 12 to 17 years. These findings have implications for decision makers, funding agencies, and child protection agencies to improve jurisdictional resources to reduce the socioeconomic vulnerabilities of reunifying families.

## 1. Introduction

The ability for parents to care for children is influenced by proximal and environmental challenges. Parents living in poverty often struggle to balance basic financial demands, such as the cost of food, accommodation, transportation, clothing, special-needs healthcare, material, and educational needs. Combined, the presence of these proximal and environmental risk factors for families in situations of poverty creates greater socioeconomic vulnerabilities that may impede family functioning and the reunification of families with placed children.

Across North America, child protection services are structured around basic legal and social tenants encouraging interventions that avoid out-of-home placement if safe to do so, but when children must be placed in out-of-home care, family reunification becomes the primary goal of intervention. However, reunification requires the resolution of multi-faceted risk factors at both the individual-level and environmental-level that result in socioeconomic vulnerability, and temporary out-of-home placement is meant to be used by parents to address the risk factors that lead to child protection service engagement. For example, in a case leading to the substantiation of neglect, a decision to reunify a family may come when parents complete support programs (e.g., parenting classes, addiction, or mental health treatment), demonstrate the use of supportive community services (e.g., food banks, employment and skills development programs), and make other efforts to improve case specific risk factors such as housing quality or security. These types of risk factors are important for child wellbeing but they are also emblematic of systemic socioeconomic vulnerabilities that are not within child protection mandates or capacities to solve [1,2]. Many jurisdictions have implemented community supports and resources to reduce stressors for socioeconomically vulnerable families and children. However, at present, these resources may not be enough to counteract the multifaceted risk factors associated with socioeconomic vulnerabilities. Previous research by Esposito and associates [3] has shown that socioeconomic vulnerabilities decrease parents’ ability to provide safe and adequate environments for their children, leading to a higher risk of out-of-home placement for children of such families. While it is well established that socioeconomic vulnerability increases the likelihood of placement, the effects of socioeconomic vulnerability on reunification is less clear, as is the extent to which the multi-faceted risk factors of vulnerable families and children vary based on the availability of jurisdictional prevention services and resources.

Socioeconomic vulnerability and its impact on family reunification has received little focused attention in the past two decades compared with the more fulsome body of work analyzing the relationship between socioeconomic vulnerability, child protection service involvement, and placement in out-of-home care, particularly as it relates to neglect and chronic stress [2,3,4,5,6,7,8,9,10,11,12,13,14,15,16,17,18]. In a previous study, Esposito and associates [19] found that neighborhood socioeconomic disadvantages, combined with individual-level and case-specific factors, were associated with a decreased likelihood of reunification. However, this previous study did not take into consideration the effects of psychosocial services offered to support child protective services—e.g., referrals for services aimed at improving parenting and family functioning—or government social service spending per child capita on mental health services, one of the primary concerns here. In general, little is also known about the extent to which psychosocial services might mitigate the influence of socioeconomic vulnerability on parents’ ability to ensure the adequately safe environments needed for family reunification. The current study seeks to fill this gap by examining the extent to which jurisdictional variations in socioeconomic vulnerability, psychosocial services, and social services spending impact reunification, after controlling for individual-level risk factors and jurisdictional latent differences in delivery of child protection services. While we expect to find jurisdictional variation in reunification based on the concentration of socioeconomically vulnerable families, the nature of the relationship between social spending, socioeconomic vulnerability, and psychosocial spending is exploratory in nature.

### Background

Poverty is defined as insufficient financial resources to meet some level of basic need [20], and the condition of poverty can be measured at the household-level. However, poverty more broadly contextualized is associated with numerous other factors that negatively affect the overall wellbeing of children and families and create socioeconomic vulnerabilities. Poverty (as measured by financial resources) have been linked to an increase in family stress [21] and vulnerabilities in health and wellbeing based on observable disadvantage [22,23]. In the child protection arena, poverty has been associated with investigation and placement, most often for neglect [7,10,11,15,24]. Beyond insufficient financial resources at the household-level, the concentration of poverty within neighborhoods [24,25,26,27], stressors related to lone parenting, the feminization of poverty and the prevalence of female-headed families amongst the poorest families [28,29,30,31], as well as the compounding effects of educational attainment, substance misuse, and poverty’s detriments to the physical and mental health of caregivers [7,31,32,33,34,35], all influence child protection decision-making.

Poverty also matters in reunification. Delfabbro and associates [36] identified poverty, which in their study was measured by the absence of housing and presence of financial problems, to be among the most consistent factors relating to delays in reunification outside of caregiver death or child abandonment. The effects of financial limitations on family reunification are also demonstrated by Wells and Guo [29,30,31], who found welfare reforms that reduced the monthly income of parents, slowed reunification. Hook and associates, [37] concluded that parents with children in out-of-home care had difficulty meeting both the demands of employment and child welfare mandates, which increased stress and added risk factors that again slowed reunification. It is possible that similar problems are faced by parents with children in out-of-home care in Quebec, as they are required to make financial contributions to their children’s out-of-home care needs [38], which may reduce the income needed to secure basic necessities, increase stress levels, and as a result, delay reunification.

Where the studies above focus on the influence of income and income-related supports, others find poverty related vulnerabilities to be of greater significance than lack of financial resources alone. Wulczyn, Chen, and Courtney [16] studied the reunification rates in and across jurisdictions comprised of female-headed, single parent, impoverished families living in racialized urban communities with high rates of children in care. The results indicated that poverty rates were insignificant for reunification, however almost every other measure, such as single parent/female-headed families, overall placement rates, and proportion of racial minority families in the population had overall slowing effect on reunification. Johnson-Reid, Drake, and Zhou [11] found that Black children reported for neglect lived in areas with a high concentration of poverty, and come from families with a longer history of poverty than White children reported for neglect, suggesting that ecological context matters in child welfare investigation. Marcenko, Lyons, and Courtney [13] provide a descriptive template for ecological risk factors beyond insufficient finances that delays reunification. The authors describe poverty as the most acute problem faced by the caregivers within the sample, however housing instability and lower rates of access to social services, as well as individual-level caregiver factors such mental health/psychiatric disorders, and recent (within 12 months) substance misuse was also found to delay reunification.

These studies provide converging evidence suggesting that while poverty matters in reunification, understanding and reducing the proximal and environmental vulnerabilities surrounding families in situations of poverty is also important in reunification efforts. However, what is not clear from prior evidence is the extent to which psychosocial services and social service spending may mitigate the likelihood of reunification among socioeconomically vulnerable families. Additionally, and as will be examined closely here, prior studies have not differentiated between the youngest children and the oldest, thereby masking age-specific clinical differences associated with reunification. The present population-based longitudinal multilevel study, therefore, makes an unique contribution by examining the extent to which jurisdictional variations in socioeconomic vulnerability, psychosocial services, and social services spending impact the likelihood of reunification, after controlling for individual-level risk factors and jurisdictional latent differences in delivery of child protection services.

## 2. Method

This study uses a multilevel longitudinal research design that draws population data from various sources: (1) longitudinal administrative data from Quebec’s child protection agencies [19,33]; (2) Canadian Census data; and, (3) social assistance, psychosocial services and social services spending data from the Ministry of Health and Social Services (MHSS). The first data source consists of anonymized longitudinal clinical administrative data extracted from a common provincial information system used by all mandated child protection jurisdictions across the province of Quebec, containing data on approximately 450,000 children dating back to 1989. All variables used in this study—except for jurisdictional-level measures of socioeconomic vulnerability, psychosocial consultations, and social services spending—were created using these clinical administrative data. The second and third data source is intra-provincial data retrieved from Census Canada and MHSS, used to create jurisdiction-specific measures of socioeconomic vulnerability, psychosocial service consultations, and social services spending. This study contains secondary analysis of data approved (CÉR CJM-IU: 14-04-02 and CJQ-IU-2013-16) for the purposes of understanding socioeconomic disadvantages and the placement trajectories of all children served by child protection in the province of Quebec.

The clinical population studied consists of all children (*N* = 39,882) placed in out-of-home care—defined as any placement lasting longer than 72 h—for the first time between 1 April 2002, and 31 March 2013, and were followed for a minimum of 20 months from their initial placement. ***Family reunification*** is the dependent variable in this study. Family reunification is defined as the first reunification of placed children and consists of a return to one or both birth parents or families of origin. The follow-up period started from the date of initial placement within a child protection jurisdiction to the date of reunification or end of follow-up period—30 November 2014—or the child’s 18th birthday, whichever came first.

### 2.1. Explanatory First and Second-Level Variables

The model includes first-level variables reflecting the ecological influences that affect the likelihood of reunification. ***Gender*** is a nominal variable with female as the reference group for male. ***Reason for placement*** consists of the following dichotomous constructs: (1) psychological and emotional abuse, which includes rejection, denigration, exposure to intimate partner violence, and exploitation; (2) physical, material, and health neglect, which includes physical neglect, medical neglect, school neglect, and material deprivation; (3) parent high-risk lifestyle, which represents parents’ lifestyle resulting in a failure to supervise or protect the child, including abandonment due to parental absence, substance abuse, refusal to assure child care, and risk of neglect; (4) confirmed and risk of physical abuse; (5) confirmed and risk of sexual abuse; (6) behavioral problems such as harming behavior, violence towards self and others, child substance abuse, school behavioral problems, runaway behavior, and destruction of property. ***Source of referral*** includes the following nominal values: (1) community health and social services clinics (CLSC); (2) child protection agency; (3) extended family and neighbors; (4) school staff; (5) police; (6) hospital staff; and, (7) other professional institutions.

Second-level measures were generated for all of Quebec’s 166 health and social service jurisdictions. ***Families with children in socioeconomic vulnerability*** is an index created to reflect the relative level of socioeconomic disadvantages, weighted by the population of children 0–17 years in each jurisdiction. The index was created using data from the 2006 Canadian Census and the 2011 National Household Survey, as well as administrative data from the Ministère du Travail, de l’Emploi et de la Solidarité Sociale on the proportion of families with children receiving social assistance payments as a last-resort source of revenue. For each of the two years, the index includes six indicators: (1) total population aged 15 years and over that are inactive or unemployed; (2) total population aged 15 years and over that do not possess a secondary school diploma; (3) median income of individuals 15 years and over; (4) median family income; and, (5) median household income. For the sixth indicator, we calculated the rate of families receiving social assistance as a last-resort source of revenue for 2006 and 2011, and then used a log base 10 transformation to normalize all data. A factorial analysis was used to create a single index of socioeconomic disadvantage. In order to identify and correct for the high child population jurisdictions, the index was then weighted for each jurisdiction based on the logistic function of the standardized number of children aged 0–17 years. The weighted factorial conversion reflects jurisdictional estimates of the concentration of families with children living in situations of socioeconomic vulnerability. The index has a minimum jurisdictional score of −1.97 representing jurisdictions with the lowest socioeconomic risk families and a maximum score of 1.48 representing the highest socioeconomic risk jurisdictions. The index has a mean score of −0.071 (s.d. 0.642) and median of 0.070. The ***rate of psychosocial service consultation*** is a compound measure reflecting jurisdictional variations in the mental health needs of the adult population of families with children. The measure reflects the average rate per thousand parents for years 2007 to 2015 for psychosocial service consultations regarding: (1) the development and social integration of children aged 0–5 years; (2) psychosocial services for troubled youth; (3) emergency services for mental health concerns; and, (4) psychosocial home visitation services. The measure has a minimum rate of 1.93 per thousand and a maximum score of 25.45 per thousand representing the highest rate jurisdiction. The average rate of parents consulting for psychosocial services in Quebec is 7.01 per thousand. The measure of ***social services spending*** consists of a compound measure of uncapped spending aggregated by jurisdiction between 2010 and 2014. This compound measure reflects the average rate per child capita spending for psychosocial, substance dependency, and community social services, excluding child welfare services spending. In order to reduce the influence of skewed extreme values usually associated to the higher cost of services in the rural remote jurisdictions in the province, we transformed the social services spending data by taking the natural logarithm (*log_e_x*) of the jurisdictional spending per child capita estimates. The normalized measure has a minimum jurisdictional spending estimate of 10.05 representing the lowest jurisdictional spending per child capita on social services and a maximum jurisdictional spending estimate of 17.81 representing the highest jurisdictional per child capita spending for social services.

### 2.2. Analytic Model

This study uses multilevel Cox proportional hazard models to estimate the individual and jurisdictional impact on the likelihood of reunification. The proportional hazard component identifies the probability of reunification at time *t* given that children were placed in out-of-home care until time *t*. Cox proportional hazard models were chosen given its less restrictive distribution assumptions. The multilevel component in this study models the variation between child protection jurisdictions. The overall statistical model is specified as:

*ln*[*H*(*t*)/*H*_0_(*t*)]*_ij_* = *b*_00_ + *b*_1_*X*_1*ij*_ ……*+ b_k_X_kij_* + *C*_1*J*_*Z*_1*j*_……+ *C*_3*J*_*Z*_3*j*_ + *U_j_* + *e_ij_*

where *X*_1*ij*_……*X_kij_* represents individual measures for children *i* in jurisdiction *j*; *Z_j_* represents second-level measures (composite estimate of families with children in socioeconomic vulnerability, rate of psychosocial service consultations, and estimate of social services spending per child capita) for jurisdiction *j*; *U_j_* is the random effect at the second-level associated with jurisdiction *j*; and *e_ij_* is the random error. The *exp*^(*b|c*)^ represents the likelihood estimate (expressed as a hazard ratio) of reunification for each variable holding all other measures constant. Given the different units of measurement, variable estimates have been standardized using both the variance of the variables and the outcome, allowing for the comparison of the relative importance of each with reunification. Interpretations of standardized exponential coefficients allow us to determine whether a change of one standard deviation in one variable produces more of a change in the probability of reunification than in another variable. Lastly, the proportion of explained jurisdiction variance was calculated as the relative difference in residual variance, where *V*_0_ represents the residual variance in the null multilevel model accounting for the nested data structure (children nested in community health and social service jurisdictions) and *V*_1_ represents the second-level residual variance once we include families with children in socioeconomic vulnerability, rate of psychosocial service consultations and social services spending per child capita (final multilevel model). The data set was constructed and transformed using SPSS version 22 and analyzed using Mplus 7. Statistical tests were conducted at 95% level of confidence.

### 2.3. Analytic Process

The analysis was performed in several steps. First, descriptive analyses were used to examine all variables and reunification (see Table 1). Table 2 presents correlational interactions between jurisdictional composite measures of families with children in socioeconomic vulnerability, psychosocial service consultations, and social services spending, respectively, and overall jurisdictional percentage of children reunified. This allowed us to assess the nature and magnitude of the bivariate interactions. Next, a null multilevel model with no jurisdictional measures was modeled in order to obtain the latent residual variance, followed by a final multilevel model with all three jurisdictional measures entered allowing for the computation of the relative difference in residual variance. Table 3, Table 4 and Table 5 report estimates of the multilevel Cox proportional hazard regression models for age-specific groups—children age 0 to 4 years (see Table 3, *N* = 10,243); children age 5 to 11 years (see Table 4, *N* = 8688); and children age 12 to 17 years (see Table 5, *N* = 20,951).

## 3. Results

A description of the clinical population appears in Table 1. The vast majority of placed children are reunified with their families. The total clinical population of children studied included 39,882 children placed in out-of-home care for the first time, of which 67.7% (*N* = 27,012) were reunified with their families. There was considerable variability across age groups. The proportion of children placed out-of-home and reunified is highest (76.8%) amongst those aged 12 to 17 at initial placement, followed by 5 to 11-year-olds (65.9%) and 0 to 4-year-olds (50.6%). There are relatively equal proportions of male and female children placed out-of-home, although males have a higher proportion of reunification. Younger and older children were also placed out-of-home for different reasons. Children younger than 12 years were placed primarily because of parents’ high-risk lifestyles and psychological and emotional abuse, while over half of children aged 12 to 17 years old were placed out-of-home for severe behavioral difficulties as the primary concern. The highest proportions of placed children were reported by a family member (25.3%), except for children 5 to 11 years old, for which slightly over one quarter (25.9%) were reported by a school staff. Irrespective of age at initial placement out-of-home, half of all placed children returned to live with their families within the first 93 days of initial placement. Median time to reunification is least (80 days) for 0 to 4-year-olds, followed by 12 to 17-year-olds (86 days), and the lengthiest for 5 to 11-year-olds (131 days).

Statistically significant correlations were found between jurisdictional reunification and second-level measures (see Table 2). At the bivariate level, we see that 20.7% (*r* = −0.455, *p* < 0.001) of the variation in jurisdictional reunification is explained by the density of families with children in socioeconomic vulnerability, 11.2% (*r* = −0.336, *p* < 0.001) by social services spending per child capita, and 3.3% (*r* = −0.183, *p* < 0.001) by the rate of psychosocial service consultations, respectively. Jurisdictions with a higher percentage of families with children in socioeconomic vulnerability, a higher rate of psychosocial service consultation, and social services spending also have lower rates of reunification. Higher per capita spending for social services was also related to a higher rate of psychosocial service consultations, as 26.5% (*r* = 0.515, *p* < 0.001) of the variation in psychosocial consultations is explained by the level of spending per child capita in social services. The correlation between families with children in socioeconomic vulnerability, psychosocial consultations, and social services spending was statistically and positively associated. Jurisdictions with a higher percentage of families with children in socioeconomic vulnerability also have a higher rates of psychosocial service consultations (*r* = 0.280, *p* < 0.001) and social services spending (*r* = 0.293, *p* < 0.001)—a possible reflection of increased need.

### 3.1. Multilevel Cox Proportional Hazard Model of Family Reunification for Children 0 to 4 Years Old

Table 3 presents the null nested and final multilevel Cox proportional hazard estimates of family reunification for children age 0 to 4 years. The null nested model produced a Log likelihood statistic of −36,973 (*df* = 8), and the final model produced a Log statistic of −35,863 (*df* = 11). The decreasing Log estimates suggest that the final multilevel model for children age 0 to 4 years is a better model fit.

Within each category, the most influential factors predicting a decreased likelihood of family reunification were: children placed because of their parents’ high-risk lifestyle (Beta = −0.597, *t* = −5.706); and, children reported by hospital staff (Beta = −0.522, *t* = −5.096). Analyzing each second-level measure independently, the concentration of families with children in socioeconomic vulnerability and rate of psychosocial service consultations were significant predictors of reunification, whereas social services spending was not statistically significant. However, under the final model, all variables were significant predictors of reunification. Controlling for higher social services spending, psychosocial services increased the likelihood of reunification while socioeconomic vulnerabilities decreased the likelihood of reunification. Combined, 24.0% of the variation in territorial reunification is explained by differences in socioeconomic vulnerability, psychosocial service consultations, and social services spending.

### 3.2. Multilevel Cox Proportional Hazard Model of Family Reunification for Children 5 to 11 Years Old

Table 4 presents the null nested and final multilevel Cox proportional hazard estimates of family reunification for children age 5 to 11 years. The null nested model produced a Log likelihood statistic of −40,353 (*df* = 10), and the final model produced a Log statistic of −40,147 (*df* = 13). The decreasing Log estimates suggest that the final multilevel model for children age 5 to 11 years is a better model fit.

The most influential factor predicting a decreased likelihood of family reunification for placed children 5 to 11 years is physical, material, school, and health neglect (Beta = −0.682, *t* = −9.281). Analyzing each second-level measure independently, the concentration of families with children in socioeconomic vulnerability and social services spending were significant predictors of decreased reunification, whereas psychosocial service consultations was not statistically significant. All variables were significant predictors of reunification in the final model. Controlling for higher social services spending, psychosocial services increased the likelihood of reunification while socioeconomic vulnerabilities decreased the likelihood of reunification. Combined, 12.5% of the variation in jurisdictional reunification is explained by differences in socioeconomic vulnerabilities, psychosocial service consultations and social services spending.

### 3.3. Multilevel Cox Proportional Hazard Model of Family Reunification for Children 12 to 17 Years Old

Table 5 presents the null nested and final multilevel Cox proportional hazard estimates of family reunification for children age 12 to 17 years. The null nested model produced a Log likelihood statistic of −10,252 (*df* = 10), and the final model produced a Log statistic of −10,149 (*df* = 13). The decreasing Log estimates suggest that the final multilevel model for children age 12 to 17 years is a better model fit.

The most influential factor predicting a decreased likelihood of family reunification for placed children 12 to 17 years parents’ high risk lifestyle (Beta = −0.390, *t* = −6.854), child protection agency (Beta = −0.265, *t* = −5.727). Analyzing each second-level measure independently, the concentration of families with children in socioeconomic vulnerability and social services spending were significant predictors of decreased reunification, whereas psychosocial service consultations was not statistically significant. While psychosocial service consultation remains non-significant in the final model, socioeconomic vulnerabilities and social services spending decreased the likelihood of reunification. Combined, 21.4% of the variation in territorial reunification is explained by differences in socioeconomic vulnerabilities, psychosocial service consultations, and social services spending.

## 4. Discussion

This population-based study uses the combination of clinical administrative child protection data for the province of Quebec, psychosocial services and social services spending data from the Ministry of Health and Social Services (MHSS) and the Canadian Census in order to examine the extent to which jurisdictional variations in socioeconomic vulnerability, psychosocial services, and social spending influence reunification. We must recognize at this point that although these findings are based in part on data derived from Quebec’s 166 health and social service jurisdictional aggregations, the population-based nature of the study reflects all families served by child protection and that the probability of reunification varies directly as a function of the needs of these families.

Our study supports findings from Esposito and colleagues [19] suggesting that the decreased probability of reunification is explained primarily by family difficulties specific to physical, material, and health neglect, and parents’ high-risk lifestyle resulting in a failure to supervise or protect the child. Building on research suggesting a decreased likelihood of reunification among parents living in situations of poverty [9,11,13,16,18,19,31,36,39], this study suggests that broader socioeconomic vulnerabilities also matter for reunification. While at the bivariate level, psychosocial service consultation decreased the likelihood of reunification, at the multilevel it increased the probability of reunification for younger children aged less than 11 years. Given this, we assume that higher psychosocial services consultations and social services spending may reflect the increased needs of socioeconomically vulnerable families, but more research is needed to understand jurisdictional variations in the availability, accessibility, and quality of family support services and resources, and the concentration of child protection engaged families.

In comparison to other Canadian jurisdictions, Quebec has heavily invested in socially progressive programs aimed at reducing child poverty and social exclusion, and yet despite these investments, this study finds that socioeconomic vulnerabilities still matters for reunification. The implication of this finding is that in jurisdictions, in Canada or elsewhere, where there is less capital and social investment on family-centered programs, socioeconomic vulnerabilities may have an even larger impact on reunification. The above-mentioned studies and the current study indicate that while financial resources are a part of the reunification picture, the stress of broader socioeconomic vulnerabilities are also at play below the surface in family reunification. Specifically, this study suggests a jurisdictional sensitivity to reunification—in that variation in socioeconomic vulnerability, accounting for per capita social services spending and psychosocial services—continues to significantly explain why some jurisdictions have lower reunification rates. The socioeconomic vulnerabilities that surround insufficient finances—stress, prevalence of single-parent households, employment, schooling, mental health and addiction, and other case-specific problems—require intervention to improve reunification rates and timeframes, particularly for the very young [40], as long-term placement has been linked to placement instability, emotional and behavioral difficulty [41,42,43], and greater public spending [43].

## 5. Limitations

While this study is unique in allowing for a provincial population-based longitudinal analysis of ecological factors that influence reunification for placed children, several limitations influence the findings. This study did not adjust for the autocorrelation that may result because of siblings. The clinical-administrative data does not allow for the identification of siblings. In addition, including children’s ethno-racial background as a predictive characteristic in the final multilevel hazard models posed a particular methodological challenge given that for 15.7% of placed children the ethno-racial information was not identified and that missing information was not random.

## 6. Conclusions

While providing socioeconomic supports are beyond the scope of child protection policy and practice, efforts to ensure that a supportive structure—integration of community partners and preventative social services needed to minimize the burden and stress load of socioeconomic vulnerabilities and increase coping and parenting skills—is in place in order to improve family functioning and children’s chances for reunification. Given the consistent findings that socioeconomically vulnerable families with lower rates of reunification tend to be clustered, increased or focused family-based supports can be directed to jurisdictional enclaves with high concentration of families living in poverty. Specifically, ensuring maximized access to community services to support child protection intervention, employment opportunities, subsidized daycare and early education for jurisdictions with the highest concentration of families in socioeconomic difficulties should be considered to reduce the impact of socioeconomic disparities of the population served. Monitoring efforts should also be made to ensure that community family support services are responding appropriately in addressing family functioning concerns, which may also assist in improving the likelihood of reunification. Failing to address the socioeconomic vulnerabilities faced by many of the families served by child protection will limit the ability to improve family circumstances, and ultimately the chances of reunifying placed children—for more on this topic see Esposito and colleagues [3] and others in the special issue on the economic causes and consequences of child maltreatment in Children and Youth Services Review.

The multilevel and longitudinal population-based nature of this analysis provides an opportunity to empirically measure what is often unavailable data at the individual-level; however, further analysis will be carried out to test the robustness of the results reported in this study. Future research will examine how changes in family policies influence the likelihood of reunification over time, as well as the stability of reunified children. We also plan to utilize geographic technologies to understand the spatial disparities in family-level socioeconomic vulnerabilities of children placed in out-of-home care and the stability of reunified children by comparing the characteristics of high and low socioeconomic disadvantaged jurisdictions. We will explore whether socioeconomically similar enclaves share ecological and endogenous characteristics (i.e., ethno-racial disparity in child protection services, variations in social spending, availability and accessibility of family-based support resources, etc.), and the ways in which those characteristics impact child and family well-being. Understanding these characteristics is critically important to proactively addressing the challenges faced by vulnerable families and improving the well-being of children.

## Figures and Tables

**Table 1:** First-level descriptive factors.

Individual Factors	Children Placed 0–17 (*N* = 39,882)	Children Placed 0–4 (*N* = 10,243)	Children Placed & Reunified 0–4	Children Placed 5–11 (*N* = 8688)	Children Placed & Reunified 5–11	Children Placed 12–17 (*N* = 20,951)	Children Placed & Reunified 12–17
**Family reunification:**	67.7% (*N* = 27,012)		50.6% (*N* =5183)		65.9% (*N* = 5728)		76.8% (*N* = 16,101)
							
**Gender:**							
Male	52.0%	52.9%	46.4%	56.7%	57.5%	49.6%	50.3%
Female	48.0%	47.1%	53.6%	43.3%	42.5%	50.4%	49.7%
							
**Reason for placement:**							
Psychological & emotional abuse	24.1%	40.5%	39.6%	27.2%	25.6%	14.8%	14.0%
Physical, material, school & health neglect	5.5%	10.5%	10.1%	7.9%	6.2%	2.1%	1.7%
Parent high risk lifestyle	18.5%	31.7%	31.1%	24.4%	26.1%	9.6%	8.6%
Behavioral problems	29.8%			5.6%	6.8%	54.4%	57.1%
Confirmed and risk of sexual abuse	4.6%	2.3%	2.3%	7.0%	6.4%	4.7%	4.3%
Confirmed and risk of physical abuse	17.5%	14.9%	16.9%	27.9%	28.9%	14.4%	14.3%
							
**Source of referral at placement:**							
Child protection agency	11.4%	13.8%	12.1%	11.6%	11.9%	11.2%	11.1%
Police	18.9%	15.9%	17.6%	17.4%	14.7%	22.7%	20.5%
Other professional institutions	8.3%	12.9%	12.8%	8.8%	8.6%	6.4%	5.7%
School	15.0%			25.9%	27.1%	18.2%	18.4%
Hospital staff	21.1%	37.9%	37.6%	15.1%	15.9%	15.5%	15.5%
Family	25.3%	19.5%	19.9%	21.2%	21.8%	26.0%	28.8%
							
**Time to reunification from initial placement:**							
Median days to reunification	93 days		80 days		131 days		86 days

**Table 2:** Correlation between jurisdictional reunification, families with children in socioeconomic vulnerability, rate of psychosocial service consultations, and social services spending per capita.

	Jurisdictional Reunification	Families with Children in Socioeconomic Vulnerability (2006–2011) (FSS)	Rate of Psychosocial Service Consultation (2007–2015) (PSC)	Social Services Spending per Child Capita (2010–2014) (SSS)
Jurisdictional reunification	1			
Families with children in socioeconomic vulnerability (2006–2011) (FSS)	−0.455 ***	1		
Rate of psychosocial service consultation (2007–2015) (PSC)	−0.183 ***	0.280 ***	1	
Social services spending per child capita (2010–2014) (SSS)	−0.336 ***	0.293 ***	0.515 ***	1

*** *p* < 0.001.

**Table 3:** Multilevel Cox proportional hazard model of family reunification for placed children age 0 to 4 years.

	Number of Events and Censored Values								
	Total	Events	Censored	% Censored					
	10,243	5183	5060	49.4%					
	Null Mode					Final Model			
**Level 1**	**Beta**	**t**	**Adj. HR**	**(95% CI)**		**Beta**	**t**	**Adj. HR**	**(95% CI)**
**Child sex**									
Male (female ref)	0.064	0.952	1.07	(0.93, 1.22)		0.072	1.078	1.07	(0.94, 1.22)
**Reason for initial placement**									
Psychological & emotional abuse	−0.213	−2.265	0.808 *	(0.67, 0.97)		−0.205	−1.831	0.815	(0.65, 1.01)
Physical, material & health neglect	−0.323	−3.430	0.724 ***	(0.60, 0.87)		−0.322	−3.437	0.725 ***	(0.60, 0.87)
Parents’ high risk lifestyle	−0.604	−5.729	0.545 ***	(0.44, 0.67)		−0.597	−5.706	0.550 ***	(0.45, 0.67)
Risk of or sexual or physical abuse (ref)									
**Source of referral**									
Child protection agency	−0.458	−5.241	0.633 ***	(0.53, 0.75)		−0.450	−5.160	0.638 ***	(0.54, 0.76)
Police	0.442	5.339	1.55 ***	(1.32, 1.83)		0.450	5.453	1.57 ***	(1.34, 1.84)
Hospital staff	−0.525	−5.124	0.592 ***	(0.48, 0.72)		−0.522	−5.096	0.593 ***	(0.49, 0.73)
Other prof. institutions	0.076	0.946	1.08	(0.92, 1.26)		0.076	0.939	1.08	(0.92, 1.26)
Family (ref)									
**Level 2**	**Null Model**	**FSS Only**	**PSC Only**	**SSS Only**		**FSS & PSC & SSS**			
Families with children in socioeconomic vulnerability (2006−2011) (FSS)		0.797 * (0.67, 0.96)				−0.244	−2.759	0.783 *** (0.66, 0.93)	
Rate of psychosocial service consultation (2007−2015) (PSC)			1.332 * (1.04, 1.70)			0.588	4.761	1.800 *** (1.41, 2.30)	
Social services spending per child capita (2010−2014) (SSS)				0.808 (0.61, 1.73)		−0.336	−3.012	0.715 *** (0.57, 0.88)	
	**Null Model**	**FSS Only**	**PSC Only**	**SSS Only**		Final Model (FSS & PSC & SSS)			
**Residual Variance (V1)**	0.045	0.041	0.043	0.043		0.034			
V explained ((V0 – V1)/V0)100		8.0%	4.0%	4.0%		24.0%			

* *p* < 0.05, ** *p* < 0.01; *** *p* < 0.001.

**Table 4:** Multilevel Cox proportional hazard model of family reunification for placed children age 5 to 11 years.

	**Number of Events and Censored Values**								
	**Total**	**Events**	**Censored**	**% Censored**					
	8688	5728	2960	34.1%					
	**Null Model**					**Final Model**			
**Level 1**	**Beta**	**t**	**Adj. HR**	**(95% CI)**		**Beta**	**t**	**Adj. HR**	**(95% CI)**
**Child sex:**									
Male (female ref)	0.034	0.402	1.04	(0.85, 1.22)		0.032	0.381	1.03	(0.87, 1.22)
**Reason for initial placement:**									
Psychological & emotional abuse	−0.236	−2.258	0.790 *	(0.64, 0.97)		−0.237	−2.275	0.789 *	(0.63, 0.97)
Physical, material, school & health neglect	−0.683	−9.323	0.505 ***	(0.44, 0.58)		−0.682	−9.281	0.506 ***	(0.44, 0.58)
Parents’ high risk lifestyle	−0.353	−3.287	0.702 ***	(0.57, 0.87)		−0.354	−3.296	0.702 **	(0.57, 0.87)
Behavioral problems	0.167	2.360	1.82 *	(1.03, 1.36)		0.164	2.328	1.18 *	(1.03, 1.35)
Risk of or sexual or physical abuse (ref)									
**Source of referral:**									
Child protection agency	−0.192	−1.557	0.825	(0.65, 1.05)		−0.189	−1.534	0.828	(0.65, 1.05)
Police	0.383	3.366	1.47 **	(1.17, 1.83)		0.387	3.407	1.47 ***	(1.18, 1.84)
Hospital staff	0.190	1.997	1.21 *	(1.01, 1.46)		0.187	1.969	1.21 *	(1.01, 1.45)
School	0.592	6.588	1.81 ***	(1.52, 2.16)		0.594	6.637	1.81 ***	(1.52, 2.16)
Other prof. institutions	0.034	0.328	1.03	(0.84, 1.27)		0.030	0.280	1.03	(0.84, 1.27)
Family (ref)									
**Level 2**	**Null Model**	**FSS Only**	**PSC Only**	**SSS Only**		**FSS & PSC & SSS**			
Families with children in socioeconomic vulnerability (2006−2011) (FSS)		0.837 * (0.70, 0.96)				−0.171	−2.280	0.843 * (0.73, 0.97)	
Rate of psychosocial service consultation (2007−2015) (PSC)			1.132 (0.87, 1.48)			0.400	2.704	1.492 ** (1.12, 1.99)	
Social services spending per child capita (2010−2014) (SSS)				0.753 * (0.59, 0.97)		−0.314	−2.663	0.731 ** (0.58, 0.92)	
	**Null Model**	**FSS Only**	**PSC Only**	**SSS Only**		**Final Model (FSS & PSC & SSS)**			
**Residual Variance (V1)**	0.048	0.045	0.048	0.046		0.042			
V explained ((V0 – V1)/V0) 100		6.3%	0%	4.2%		12.5%			

* *p* < 0.05, ** *p* < 0.01; *** *p* < 0.001.

**Table 5:** Multilevel Cox proportional hazard model of family reunification for placed children age 12 to 17 years.

	**Number of Events and Censored Values**								
	**Total**	**Events**	**Censored**	**% Censored**					
	20,951	16,101	4850	23.1%					
	**Null Model**					**Final Model**			
**Level 1**	**Beta**	**t**	**Adj. HR**	**(95% CI)**		**Beta**	**t**	**Adj. HR**	**(95% CI)**
**Child sex:**									
Male (female ref)	−0.052	−1.330	0.949	(0.44, 2.04)		−0.052	−1.336	0.946	(0.88, 1.02)
**Reason for initial placement:**									
Psychological & emotional abuse	−0.143	−2.538	0.867 **	(0.78, 0.97)		−0.142	−2.523	0.868 *	(0.78, 0.97)
Physical, material, school & health neglect	−0.263	−5.652	0.769 ***	(0.70, 0.84)		−0.264	−5.652	0.768 ***	(0.70, 0.84)
Parents’ high risk lifestyle	−0.390	−6.847	0.677 ***	(0.61, 0.76)		−0.390	−6.854	0.677 ***	(0.61, 0.76)
Behavioral problems	0.511	8.242	1.67 ***	(1.48, 1.88)		0.511	8.248	1.67 ***	(1.47, 1.88)
Risk of or sexual or physical abuse (ref)									
**Source of referral:**									
Child protection agency	−0.264	−5.709	0.768 ***	(0.70, 0.84)		−0.265	−5.727	0.767 ***	(0.70, 0.84)
Police	0.209	4.212	1.23 ***	(1.12, 1.35)		0.208	4.199	1.23 **	(1.12, 1.36)
Hospital staff	−0.038	−0.881	0.962	(0.88, 1.05)		−0.040	−0.929	0.961	(0.88, 1.05)
School	0.056	1.145	1.06	(0.96, 1.16)		0.056	1.136	1.06	(0.96, 1.16)
Other prof. institutions	−0.247	−5.408	0.781 ***	(0.71, 0.86)		−0.248	−5.419	0.780 ***	(0.71, 0.85)
Family (ref)									
**Level 2**	**Null Model**	**FSS Only**	**PSC Only**	**SSS Only**		**FSS & PSC & SSS**			
Families with children in socioeconomic vulnerability (2006−2011) (FSS)		0.698 *** (0.61, 0.80)				−0.314	−4.763	0.731 *** (0.64, 0.83)	
Rate of psychosocial service consultation (2007−2015) (PSC)			0.862 (0.70, 1.06)			0.156	1.177	1.69 (0.90, 1.51)	
Social services spending per child capita (2010−2014) (SSS)				0.804 * (0.67, 0.96)		−0.281	−2.745	0.755 ** (0.62, 0.92)	
	**Null Model**	**FSS Only**	**PSC Only**	**SSS Only**		**Final Model (FSS & PSC & SSS)**			
**Residual Variance (V1)**	0.056	0.047	0.055	0.054		0.044			
V explained ((V0 – V1)/V0) 100		16.1%	1.8%	3.6%		21.4%			

* *p* < 0.05, ** *p* < 0.01; *** *p* < 0.001.
